# Metagenomic analysis of the microbial communities and associated network of nitrogen metabolism genes in the Ryukyu limestone aquifer

**DOI:** 10.1038/s41598-024-54614-8

**Published:** 2024-02-22

**Authors:** Rio Maruyama, Ko Yasumoto, Nanami Mizusawa, Mariko Iijima, Mina Yasumoto-Hirose, Akira Iguchi, Oktanius Richard Hermawan, Takahiro Hosono, Ryogo Takada, Ke-Han Song, Ryuichi Shinjo, Shugo Watabe, Jun Yasumoto

**Affiliations:** 1 Kitasato University School of Marine Biosciences, 1-15-1 Kitasato, Minami, Sagamihara, Kanagawa 252-0373 Japan; 2 National Institute of Advanced Industrial Science and Technology (AIST), Tsukuba Central 7, 1-1-1 Higashi, Tsukuba, Ibaraki 305-8567 Japan; 3 Tropical Technology Plus, 12-75 Suzaki, Uruma, Okinawa 904-2234 Japan; 4 Department of Earth and Environmental Science, Graduate School of Science and Technology, Kumamoto University, 2-39-1, Kurokami, Chuo-ku, Kumamoto 860-8555 Japan; 5 Faculty of Advanced Science and Technology, Kumamoto University, 2-39-1 Kurokami, Chuo-ku, Kumamoto 860-8555 Japan; 6 International Research Organization for Advanced Science and Technology, Kumamoto University, 2-39-1 Kurokami, Chuo-ku, Kumamoto 860-8555 Japan; 7 Center for Strategic Research Project, University of the Ryukyus, Nishihara, Senbaru, Okinawa 903-0213 Japan; 8 Department of Physics and Earth Sciences, University of the Ryukyus, 1 Senbaru, Nishihara, Okinawa 903–0213 Japan; 9 Research Institute for Humanity and Nature, 457-4 Motoyama, Kamigamo, Kita-ku, Kyoto 603-8047 Japan; 10 Faculty of Agriculture, University of the Ryukyus, 1 Senbaru, Nishihara, Nakagami, Okinawa 903-0213 Japan

**Keywords:** Microbiology, Environmental sciences

## Abstract

While microbial biogeochemical activities such as those involving denitrification and sulfate reduction have been considered to play important roles in material cycling in various aquatic ecosystems, our current understanding of the microbial community in groundwater ecosystems is remarkably insufficient. To assess the groundwater in the Ryukyu limestone aquifer of Okinawa Island, which is located in the southernmost region of Japan, we performed metagenomic analysis on the microbial communities at the three sites and screened for functional genes associated with nitrogen metabolism. 16S rRNA amplicon analysis showed that bacteria accounted for 94–98% of the microbial communities, which included archaea at all three sites. The bacterial communities associated with nitrogen metabolism shifted by month at each site, indicating that this metabolism was accomplished by the bacterial community as a whole. Interestingly, site 3 contained much higher levels of the denitrification genes such as *narG* and *napA* than the other two sites. This site was thought to have undergone denitrification that was driven by high quantities of dissolved organic carbon (DOC). In contrast, site 2 was characterized by a high nitrate-nitrogen (NO_3_-N) content and a low amount of DOC, and this site yielded a moderate amount of denitrification genes. Site 1 showed markedly low amounts of all nitrogen metabolism genes. Overall, nitrogen metabolism in the Ryukyu limestone aquifer was found to change based on environmental factors.

## Introduction

Groundwater is one of the major sources of drinking and agricultural water in regions where population density is high and economic activities such as industrial agriculture are well developed, even when surface water resources are sufficiently available^1,2^. Groundwater is an especially important water source in limestone areas. The Okinawa-Jima area of Japan as well as the remote island areas nearby are composed of limestone and have groundwaters with higher flow rates than river water; in fact, underground dams have been constructed to secure stable sources of irrigation water^3,4^. Groundwater is also a major water resource in other island countries around the world^5,6^.

In recent years, groundwater contamination has become a serious problem in many areas of the world. Various substances have been identified as contaminants, and despite the implementation of various control measures, contamination still occurs. Problems associated with nitrate-nitrogen (NO_3_-N) contamination are particularly severe in areas where livestock production is a major industry^7–11^. NO_3_-N contamination is thought to be caused by fertilizer application to agriculture fields^12–15^ or to originate from sewage from swine and cattle barns, as well as from livestock manure^16^. Continued exposure to groundwaters containing high levels of NO_3_-N is harmful to the human body^17^. Measures have been taken to reduce the amount of NO_3_-N in groundwater, and drinking water standards have been established in every country. However, the current level of pollution has yet to be evaluated, and a fundamental solution has yet to be reached.

Elevated NO_3_-N concentrations present groundwater contamination challenges in the southwestern islands of Japan, including the main island of Okinawa. The southern part of Okinawa Island near Naha City, Okinawa Prefecture, consists of a field cropping area, and groundwater has long been used for agricultural and domestic purposes in this region. Under the National Government's Southern Okinawa Main Island Agricultural Water Conservancy Project (1992–2005)^18^, two underground dams were constructed at Komesu and Giza to secure water for agriculture and develop water resources, and the groundwater has been used for agricultural purposes and drinking water^19–21^.

Studies have demonstrated that NO_3_-N is consumed by denitrification and nitrate reduction; these functions are carried out by microbial nitrogen metabolism and lead to a decrease in NO_3_-N concentration^22,23^. Microbial communities play particularly important roles in primary nitrogen metabolism and circulation^24,25^. Such functions have been ascertained in soil, water, and activated sludge environments, and some responsible microbial species have been identified^26–29^.

For microorganisms that have the abovementioned water purification functions, 16S rRNA amplicon analysis combined with functional gene analysis by real-time PCR has been actively employed^30,31^. For example, Guo et al.^30^ adopted metagenomic methods to identify microbial communities and functions for removing nitrogen and phosphorus from activated sludge. The bacterial communities and denitrification gene (*nirS*/*nirK*) expression in the groundwater of limestone islands have also been analysed by utilizing PCR-denaturing gradient gel electrophoresis (DGGE) and real-time PCR methods^32^. However, a comprehensive analysis of the microbial communities and associated nitrogen metabolism has not yet been conducted. We performed periodic shotgun metagenomic surveys in an urban river, the Tama River, and an enclosed bay, the Ofunato Bay, to comprehensively analyse microbial communities and to clarify the relationship between microbial functional genes and environmental factors^33–36^.

In this study, the microbial community of the groundwater and functional genes involved in nitrogen metabolism, such as the nitrate reduction, nitrogen fixation, ammonia oxidation, and denitrification genes, were analysed by the 16S rRNA amplicon and shotgun metagenomics analyses, respectively. The results revealed that nitrogen metabolism genes, mainly those associated with denitrification, were abundant in areas with high microbial diversity.

## Materials and methods

### Sample collection

**Figure 1:**
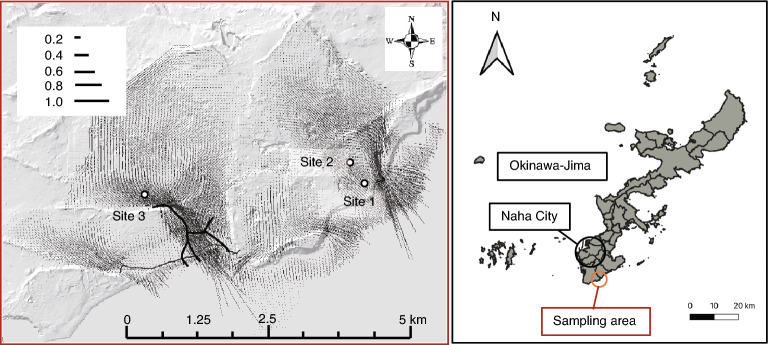
Map showing three groundwater sampling locations sites 1–3. The left panel indicates site 1, where samples were collected from a faucet at the water plant, and sites 2 and 3, where samples were collected from groundwater wells. The left panel also indicates groundwater velocities, whereas the right panel indicates the sampling area in Okinawa Island.

Yaese Town and Itoman City in the southern region of Okinawa-Jima Island were selected as the study areas. The groundwater was sampled at three sites in this area in November and December 2021 and January 2022 (Fig. 1). The water sample at site 1 was collected from a faucet at the water plant. The water samples at sites 2 and 3 were collected with a motorized water sampling device, GEO-pump-Bennett-1400 (GEO Science Laboratory, Nagoya, Japan), from observation wells (with strainers placed down to the base rock), and the groundwater levels were measured with a groundwater level meter ML50 M (Sanyo Measuring Tools Co., Ltd., Tokyo, Japan). The water samples were transported to the laboratory of the Graduate School of Engineering and Science, University of the Ryukyus, in sterilized 1 L glass containers that were kept at a low temperature, and the samples were filtered with a vacuum manifold QIAvac 24 Plus (Qiagen GmbH, Hilden, Germany) in the laboratory through a SterivexTM HV 0.22 µm filter unit (Millipore, Darmstadt, Germany). The filters were stored at − 80 °C until DNA extraction.

### Methods for measuring various environmental factors

Water temperature, pH, electrical conductivity (EC), dissolved oxygen (DO), and oxidation‒reduction potential (ORP) were measured onsite using a portable pH meter (Horiba D-54 and D-55, Horiba, Tokyo, Japan). Bicarbonate ions (HCO_3_^−^) were also measured onsite by an alkalinity titration method using an AL-DT digital titrator (HACH Company, Loveland, CO, USA). The following analyses were carried out in the laboratory. Cations and anions were determined with an Aquion Ion chromatography system (Thermo Fisher Scientific, Waltham, MA, USA), and metal elements were analysed by inductively coupled plasma‒mass spectrometry (ICP‒MS) (X-Series II, Thermo Fisher Scientific). Dissolved organic carbon (DOC) was analysed using a total organic carbon analyser (TOC-L Analyser, Shimadzu, Kyoto, Japan). Suspended solids (SS) were measured using a filtration system equipped with a glass filter (Grade CF/B, Whatman, Little Chalfont, UK), and filter was dried at 105–110 °C, according to the method of Japanese Industrial Standard (JIS) K0102. The DO values of the groundwater were only available for November 2021 at the three sites in this study because the portable pH meter equipped with the DO sensor did not work in December 2021 and January 2022.

### DNA extraction

The cells trapped on the filters were frozen, stored and subjected to DNA extraction after thawing using a DNeasy Power Water Sterivex Kit (Qiagen GmbH) at School of Marine Biosciences, Kitasato University, according to the manufacturer’s instructions. DNA concentrations were quantified with a Qubit dsDNA HS assay kit (Invitrogen, Carlsbad, CA, USA) and read with a Qubit Fluorometer (Invitrogen).

### 16S rRNA amplicon analysis

The DNA extracts prepared for different sampling months and sites were diluted to 5 ng/µL to use as a PCR template. PCR primers, 530F (5′-ACACTCTTTCCCTACACGACGCTCTTCCGATCT-NNNNNGTGCCAGCMGCCGCGG-3′) and 907R (5′-GTGACTGGAGTTCAGACGTGTGC-TCTTCCGATCTNNNNNCCGTCAATTCMTTTRAGTTT-3′)^37^, adapted with dual-index barcodes for Illumina MiSeq, were used to amplify the region spanning V4 and V5 of the 16S rRNA gene. Samples prepared for different sampling months and sites were pooled and sequenced with an Illumina MiSeq using a MiSeq Reagent Kit v3 (600 cycles) (Illumina). The amplicon metagenomic reads thus obtained have been deposited into the DNA Data Bank of Japan (DDBJ) Sequence Read Archive under the accession number DRA017557.

The amplicon reads were firstly combined by overlapping forwards and reverse reads using FLASH software (version 1.2.10, https://ccb.jhu.edu/software/FLASH/)^38^ (minimum overlap = 10; maximum overlap = 65; maximum mismatch density = 0.25; allow outie pairs = false; cap mismatch quals = false; combiner threads = 20; input format = FASTQ, phred_offset = 33; output format = FASTQ, phred_offset = 33). The obtained data were further processed by using Seqkit (version 0.5.5 https://bioinf.shenwei.me/seqkit/)^39^ to remove the Illumina sequencing adapters. By adjusting the low-quality filter using the FASTX-toolkit (version 0.6.6 http://hannonlab.cshl.edu/fastx_toolkit)^40^, the reads, which had 20% or more bases with a quality score ≥ 20, were retained. Reads of < 50 bp (*P error* limit = 0.05; Q score = 30) were trimmed, and individual reads were paired together to sequence both ends of the fragment quality-control pass. Taxonomical assignment was performed by applying 100,000 reads of each sample obtained to the SILVAngs platform (version 1.9.10/1.4.9, SILVA: r138.1)^41–44^ with an estimation of metagenomes composed of microbes not included in the database with parameter “–unclassified_estimation”.

### Shotgun metagenomic sequencing analysis

DNA libraries for shotgun metagenomics sequencing were prepared using the NextraXT DNA Preparation Kit (Illumina, San Diego, CA, USA). The quality and size of the products were assessed with an Agilent 2100 Bioanalyzer (Agilent Technologies, Santa Clara, CA, USA). Finally, 0.2 µg of each of the DNA libraries prepared was sequenced with an Illumina MiSeq using MiSeq Regent Kit v3 (600 cycles) (Illumina). The whole genome shotgun (WGS) reads thus obtained have been deposited into the DDBJ Sequence Read Archive under the accession number DRA015527.

The WGS reads were subjected to fastp (version 0.23.4)^45^ to remove low-quality reads, N-containing reads, and adapters with the following parameters: “–qualified_quality_phred 20 and –n_base_limit 20”. This process yielded standard clean data (defined here as “standard data”), and subsequently this dataset was de novo assembled using MEGAHIT (version 1.2.9)^46^ with the *k*-min of 21, *k*-max of 141 and *k*-step of 12, thus producing the contig data. The metagenome assembly was evaluated by using QUAST (version5.0.2)^47^. Gene annotation was carried out with blastn analyses^48^ using Prokka (version1.14.6)^49^ and Prodigal (version2.6.3)^50^ based on the all contig data obtained for different months and sampling sites. Transcripts per kilobase million (TPM)^51^ values were calculated by Salmon (version1.10.2)^52^. All contig data were binned into metagenome assembled genomes (MAGs) with MetaWRAP^53^ in Portable pipeline^54^ and subjected to the depiction of heatmap. The assembled MAGs were visualized by Anvi’o (version 2.2.2)^55^. The bins thus obtained were subjected to the calculation of completeness and contamination using CheckM (version 1.1.3)^56,57^. Furthermore, GTDB-Tk (version 2.3.2) was also used for assigning taxonomic classification to bacterial and archaeal genomes based on the Genome Database Taxonomy GTDB (R214)^58^.

We calculated the specific abundances of nitrogen metabolism genes per L groundwater based on the DNA concentration of groundwater as shown in following equation:

$${\text{specific}}\;{\text{abundance}}\;{\text{of}}\;{\text{nitrogen}}\;{\text{metabolism}}\;{\text{gene}}\, = \,\left( {{\text{TPM}}} \right)\, \times \,({\text{amount}}\;{\text{of}}\;{\text{DNA}} - {\text{extracted}}\;\left( {{\text{ng}}} \right){\text{/L-groundwater}}).$$

Alternatively, the WGS reads were also subjected to taxonomical analysis using the same procedures described above for the 16S rRNA amplicon datasets, except that the SILVAngs platform (version 1.9.10) was replaced by MetaPhlAn 4^59^ (version 4.0).

### Statistical analysis

Nonmetric multidimensional scaling (NMDS) analysis was performed using R software^60^ (version 4.2.0, https://cran.r-project.org/bin/windows/base/) to compare the similarity of the microbial community between different samples. The NMDS plots were generated using the Bray‒Curtis dissimilarity distance matrix. The vegan package^61^ was used, and csv files of the results of the analysis using the MEGAN database were used for the microbial community data. The correlation between the microbial communities and environmental factors was calculated using “envfit” (vegan). To examine the relationship between microbial community composition and environmental factors, distance based redundancy (dbRDA)^62^ analysis was also performed using “dbrda” (vegan). The multicollinearity among environmental factors was determined using a standard function in R.

Microbial community diversity was determined by the Shannon diversity index^63^ and Simpson diversity index^64^ using R software (version 4.2.0) “renyi” (vegan) at the genus level to examine alpha diversity. Chao I^65^ and Abundance-based Coverage Estimator (ACE)^66^ were also determined using R software (version 4.2.0) “estimate” to determine alpha diversity at the genus level.

## Results

### Environmental factors in the groundwater samples collected from three sites in the Ryukyu limestone aquifer

Table 1 shows the analytical data for environmental factors in the groundwater samples collected from three sites in the Ryukyu limestone aquifer. Site 3 was characterized by a shorter distance from the ground surface (2.4 m) than at site 2 (18.3 m), whereas the sample at site 1 was collected from a faucet at the water plant (original depth, 34.5 m). The concentrations of NO_3_-N averaged over three months at sites 1, 2 and 3 were 8.75 ± 0.46, 22.7 ± 1.75 and 6.89 ± 2.80 mg/L (mean ± SD), respectively. The water temperature, EC, HCO_3_^−^, and concentrations of Cl^−^, NO_3_-N, SO_4_^2−^, Na^+^, Mg^2+^ and Ca^2+^ were low in samples collected in December 2021 and January 2022 at site 3. Unfortunately, DO could not be measured in December 2021 and January 2022 due to equipment failure. The concentration of DO at site 3 was very low in November 2021, at less than 2 mg/L.

**Table 1:** Summary of geochemical parameters in groundwater samples collected from the three sites in the Ryukyu limestone aquifer in November and December 2021, and January 2022.

Sampling site	Collection date	Groundwater level (m)	Water temperature (°C)	pH	EC (ms/cm)	ORP (mV)	DO	DOC	HCO_3_^−^	SS	T-P	PO4-P	Cl	NO3-N	SO_4_^2−^	Na+	K+	Mg^2^+	Ca^2^+
							(mg/L)												
Site 1	Nov. 2021	34.5	23.6	7.66	0.725	184	5.02	0.274	227	10.2	0.03	0.03	52.3	9.1	71.3	35.5	2.5	9.7	87.6
	Dec. 2021	34.5	23.6	6.82	0.723	197		0.857	244.1	14.6	0.02	0.02	53.5	9.1	67.8	34.8	2.6	9.7	73.8
	Jan. 2022	34.5	23.4	7.08	0.678	198		1.057	251.4	0.0	0.02	0.02	47.8	8.1	58.6	31.5	2.8	8.5	105.1
	mean ± SD	34.5 ± 0	25.3 ± 0.09	7.19 ± 0.35	0.709 ± 0.02	193 ± 6.37		0.729 ± 0.33	240.8 ± 10.2	8.26 ± 6.12	0.02 ± 0.04	0.02 ± 0.004	51.2 ± 2.45	8.75 ± 0.46	65.9 ± 5.36	33.9 ± 1.74	2.6 ± 0.12	9.3 ± 0.57	88.8 ± 12.8
	Nov. 2021	18.3	24.4	6.8	0.919	221	5.13	1.610	263.6	0.6	0.02	0.02	56.8	20.4	110.5	48.7	4.1	21	103.3
Site 2	Dec. 2021	18.3	23.9	6.88	0.931	194		0.766	253.8	21.5	0.02	0.01	59.5	23.0	109.5	47.8	4.2	21.3	105.6
	Jan. 2022	18.3	24.1	7.03	0.976	241		0.191	253.8	0.0	0.02	0.01	58.2	24.6	109.7	47.7	4.4	20.5	127.8
	mean ± SD	18.3 ± 0	24.1 ± 0.10	6.9 ± 0.1	0.942 ± 0.02	218 ± 19.3		0.856 ± 0.58	257 ± 4.62	7.37 ± 10	0.02 ± 0	0.01 ± 0.004	58.2 ± 1.10	22.7 ± 1.75	109.9 ± 0.43	48.0 ± 0.45	4.2 ± 0.12	20.9 ± 0.33	112 ± 11
	Nov. 2021	3.19	24.2	6.68	0.861	171	1.06	2.274	331.9	41.4	0.17	0.11	48.9	10.4	98.1	30.8	8.8	17.2	143.5
Site 3	Dec. 2021	2.68	21.1	7.25	0.392	208		1.612	158.7	21.4	0.17	0.09	29.6	6.8	33.5	13.6	8.1	6.9	50.0
	Jan. 2022	1.19	20.3	7.4	0.369	230		0.249	148.9	10.7	0.21	0.14	23.4	3.5	26.3	11.3	5.8	6.0	48.7
	mean ± SD	2.35 ± 0.84	21.9 ± 1.68	7.11 ± 0.31	0.540 ± 0.23	203 ± 24.3		1.378 ± 0.84	213 ± 84.1	24.5 ± 12.7	0.18 ± 0.02	0.11 ± 0.02	34.0 ± 10.9	6.89 ± 2.80	52.6 ± 32.3	18.6 ± 8.7	7.6 ± 1.28	10.0 ± 5.08	80.7 ± 44.4

*EC* electrical conductivity, *ORP* oxidation reduction potential, *DO* dissolved oxygen, *DOC* dissolved organic carbon, *SS* suspended substance, *T-P* total phosphorus, *PO*_*4*_*-P* dissolved phosphorus, NO_3_-N nitrate nitrogen.

### Microbial communities in the groundwater samples

The number of the 16S rRNA amplicon and WGS reads, groundwater sampling volume (= filtration volume), and DNA yield per L groundwater for each sample are shown in Table 2. Site 1 had the lowest DNA yield followed by sites 2 and 3 in the increasing order. Details of 16S rRNA amplicon and WGS reads are shown in Supplementary Tables S1 and S2.

**Table 2:** The number of the 16S rRNA amplicon and WGS reads togerther with water volumes for DNA yields obtained from groundwater samples collected from the three sites in the Ryukyu limestone aquifer in November and December 2021, and January 2022.

Sampling site	Collection date	16S rRNA amplicon reads	WGS reads	Filtration volumes (L)	DNA yield (ng/L)
Site 1	Nov. 2021	378,967	1,038,312	1	62
	Dec. 2021	363,567	1,320,792	1	46
	Jan. 2022	457,837	1,544,282	1	50
	Nov. 2021	507,514	818,060	1	263
Site 2	Dec. 2021	339,797	983,092	1	531
	Jan. 2022	373,299	1,387,233	0.94	450
	Nov. 2021	416,879	1,257,735	1	849
Site 3	Dec. 2021	373,584	1,219,300	0.2	3,170
	Jan. 2022	457,837	1,220,215	0.28	1,993

*WGS* whole genome shotgun.

**Figure 2:**
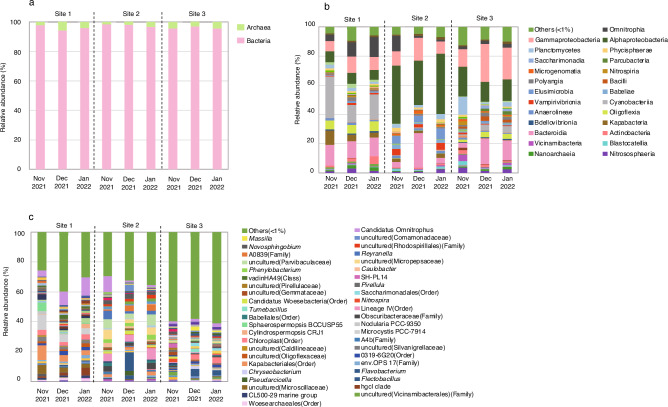
The relative abundances of microbes annotated for the 16S rRNA amplicon datasets at the domain (**a**), class (**b**) and genus (**c**) levels for groundwater samples collected from the Ryukyu limestone aquifer in November and December 2021 and in January 2022. “Others (< 1%)” indicates those with relative abundances less than 1%. Refer to the legends of Fig. 1 for the three sampling sites. Only taxonomic groups in the class and genus levels with relative abundances more than > 2.0% are listed.

Figure 2a shows the microbial communities at the domain level based on the 16S rRNA amplicons datasets. Bacteria accounted for 94.3 to 98.3% and archaea for 1.7 to 5.7%, indicating that bacteria constituted the majority of the microbial communities. As shown in Fig. 2b, classes Cyanobacteriia, Oligoflexia and Kapabacteria accounted for 12.0 to 28.5%, 5.7 to 6.7%, and 3.3 to 9.9%, respectively, at site 1. At site 2, classes Alphaproteobacteria, Elusimicrobia and Vamprivibrionia accounted for 30.2 to 41.1%, 4.5 to 7.5%, and 2.4 to 4.7%, respectively. At site 3, classes Planctomycetes and Vicinamibacteria were dominant and accounted for 12.1% and 2.3 to 4.5%, respectively, in November 2021, but these bacteria were not found in other samples. As shown in Fig. 2c, genus *Microcystis* accounted for 4.2 to 10.4% at site 1, whereas lineage IV belonging to Elusimicrobia and others (< 1%) accounted for 4.5 to 7.5% and 29.4 to 35.4%, respectively, at site 2. Site 3 was characterised by unidentified bacteria more abundant than those at the other two sites (Fig. 2c).

MetaPhlAn 4 analysis of the WGS reads revealed that unclassified reads accounted for 74 to 100% irrespective of the three sites (Supplementary Figs. S1a, S1b and S1c). While unclassified reads accounted for 97.8 to 100% at sites 2 and 3, site 1 had the highest hit among the three sites, where the most hits belonged to phylum Cyanobacteria or domain Bacteria.

### Diversity of microbial communities obtained by 16S rRNA amplicon analysis

The diversity indices of microbial communities obtained by the 16S rRNA amplicon analysis for the samples from the groundwater at site 3 were extremely high in December 2021 and January 2022 (Table 3), whereas those from site 1 were markedly low in November 2021, irrespective of Shannon, Simpson, Chao I and ACE. It was noted that Shanonn’s and Simpson’s diversity indices at site 3 in November 2021 were higher than those at sites 1 and 2.

**Table 3:** Summary of the diversity indices for the microbial communities obtained by the 16S rRNA amplicon analysis for groundwater samples collected from three sites in November and December 2021, and January 2022.

Sampling site	Collection date	Shannon's diversity index	Simpson's diversity index	Chao I's diversity index	ACE diversity index
Site 1	Nov. 2021	79.9	27.7	1305	1311
	Dec. 2021	173.9	52.6	1627	1618
	Jan. 2022	97.9	33.7	1292	1311
	Nov. 2021	81.2	31.1	1159	1149
Site2	Dec. 2021	106.9	34.1	1403	1376
	Jan. 2022	111.4	42.1	1292	1267
	Nov. 2021	273.6	132.5	1851	1818
Site3	Dec. 2021	269.1	106.2	1952	1890
	Jan. 2022	332.2	137.3	1957	1932

### Relationship between microbial communities and environmental factors

**Figure 3:**
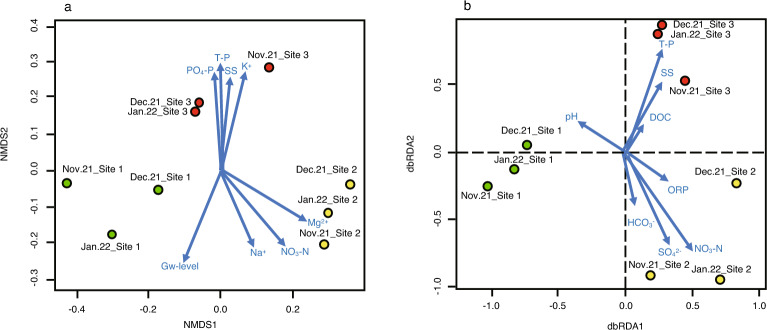
The relationship between microbial communities based on the 16S rRNA amplicon datasets and environmental parameters in the groundwater samples were collected from the three sites in November and December 2021 and in January 2022. Panel a shows the results of nonmetric multidimensional scaling (NMDS) analysis using all environmental factors listed in Table 1. Panel b shows the result of distance-based redundancy analysis (dbRDA), where environmental factors adopted were pH and ORP, and concentrations of HCO_3_^−^, DOC, SS, T-P, NO_3_-N and SO_4_^2−^, showing low collinearity (Table 3). Green, yellow and red circles indicate the microbial communities at sites 1, 2 and 3, respectively.

Figure 3a shows the results of the NMDS analysis, which was conducted to verify the relationship between the microbial communities based on the 16S rRNA amplicon datasets and various environmental factors. The plots of the microbial communities were clustered depending on the sites. In particular, the microbial communities at site 3 in December 2021 and January 2022 were highly similar to each other. When we adopted all 17 environmental factors shown in Table 1 to the NMDS analysis, several environmental factors including groundwater level, SS, T-P, PO_4_-P, NO_3_-N, Na^+^, K^+^ and Mg^2+^were found to have significant relationship with the microbial communities. The microbial communities at site 3 are correlated with PO_4_-P, T-P, SS and K^+^, whereas those at site 1 are correlated with Mg^2+^ and NO_3_-N. It seems that the groundwater level and Na^+^ have correlation with both site 1 and site 2. Then, we focused on 8 environmental factors including pH, ORP, DOC, HCO_3_^−^, SS, T-P, NO_3_-N and SO_4_^2−^, which showed low multicollinearity (Supplementary Table S3). As shown in Fig. 3b, the dbRDA analysis revealed that all 8 environmental factors mentioned above had significant relationship with the microbial communities. Especially, T-P and SS show high correlation with the microbial communities at site 3 irrespective of three months, and SO_4_^2−^ and NO_3_-N with those at site 2.

### The abundances of nitrogen metabolism genes based on all contig dataset obtained from shotgun metagenomics

**Figure 4:**
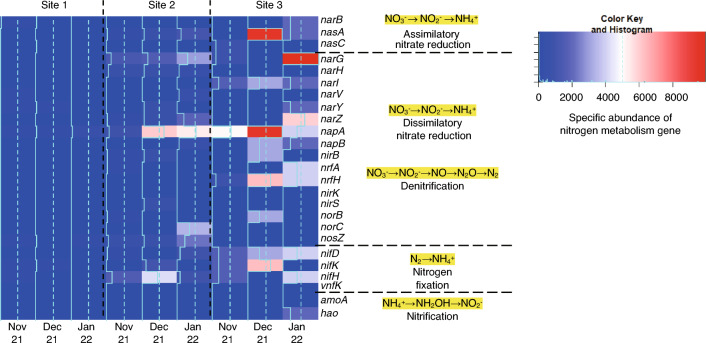
The heatmap for specific abundances of nitrogen metabolism genes with groundwater samples collected from the three sites in November and December 2021 and in January 2022. The biological processes of nitrogen metabolisms are arranged according to the oxidation states of nitrogen. The calculation for the specific abundances of nitrogen metabolism genes is detailed in the methods section and involves multiplying the TPM by the amount of DNA extracted per L of groundwater (ng/L).

Figure 4 shows the specific abundances of nitrogen metabolism genes based on the all contig dataset obtained from the shotgun metagenomes using MEGAHIT and DNA -yield per L of groundwater at the three sites in November and December 2021 and in January 2022. These genes are arranged from in descending order for dissimilatory nitrate reduction, assimilatory nitrate reduction, denitrification, nitrogen fixation, and nitrification. Twenty-five genes were found to be involved in nitrogen metabolism in the collected samples. The genes involved in dissimilatory nitrate reduction, denitrification and nitrogen fixation were almost absent at site 1, whereas the abundances at site 3 were significantly higher for all genes than those at sites 1 and 2. At site 3, *nasA* participating in assimilatory nitrate reduction and *napA* in denitrification were highly abundant in December 2021. The abundance of *narG* was also markedly high at site 3 in January 2022. It was noted that the abundances of nitrogen metabolism genes in November 2021 were much lower than those in December 2021 and January 2022 irrespective of sites 2 and 3.

### Binning of shotgun metagenomics datasets into MAGs and the distribution of nitrogen metabolism genes

**Figure 5:**
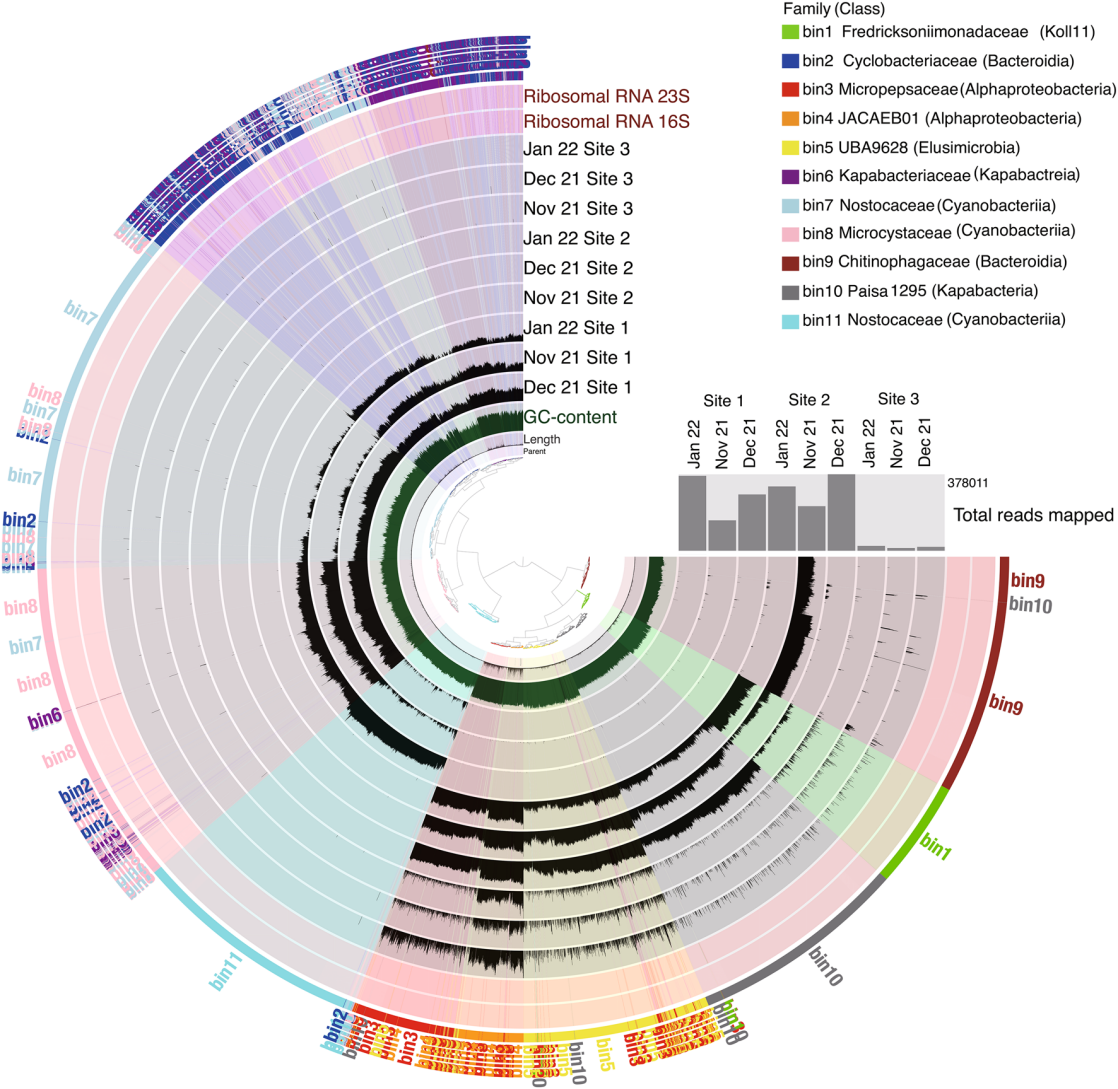
Anvi'o bin collection representation for the contig datasets obtained by shotgun metagenomics into MAGs with MetaWRAP for groundwater samples collected from the three sites in the three months. Layers from inside out include following: (1) tree displays the hierarchical clustering by the co-assembly of the contigs from draft genomes of 9 samples we determined. (2) The length that shows the actual length of a given split. (3) The GC-content. (4)–(12). The view layers display the “mean coverage” of each bin in 9 samples from the metagenomic dataset. (13) and (14) The view layers display each bin for the ribosomal RNAs identified from the metagenomic dataset. The two most outer layers show bin annotation (15) and (16). The dendrogram in the inlet shows the total reads mapped from each sample to the assembly.

**Figure 6:**
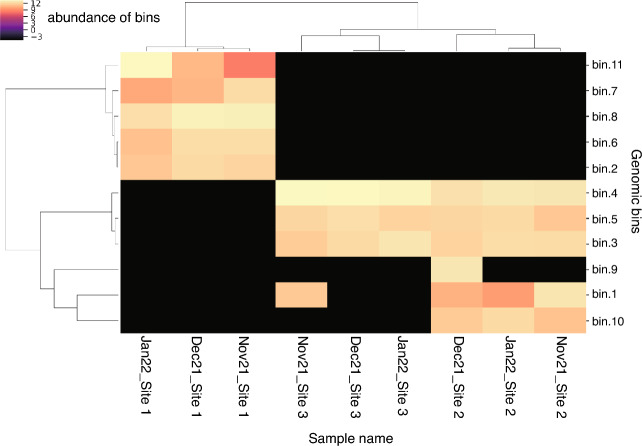
The heatmap of bin abundance estimated from MAGs with MetaWRAP for samples collected from the three sites in the three months.

Figure 6 shows the results of binning all contigs obtained from shotgun metagenomics into MAGs with MetaWRAP, which was further visualized by Anvi’o (version 2.2.2) (Fig. 5). Furthermore, CheckM and GTDB-Tk were used for checking the genomic features and assigning taxonomic classification (Table 4) and assigned results are shown in Fig. 5. Totally 11 bins were constructed, where bins 2, 6, 7, 8 and 11 were abundant at site 1, but almost absent at sites 2 and 3 (Fig. 6). Bins 2 and 6 corresponded to family Cyclobacteriaceae belonging to class Bacteroidia and family Kapabacteriaceae belonging to class Kapabacteria, respectively, whereas bins 7 and 11 both corresponded to family Nostocaceae belonging to class Cyanobacteriia but with different lineages (Table 4). Although bin 8 corresponded to family Microsystaceae, it also belonged to class Cyanobacteriia. Bins 3 and 4, which corresponded to family Micropepsaceae and undefined family JACAEB01, respectively, both belonged to class Alphaproteobacteria and were found only at site 2 and 3 irrespective of the three months as in the case of bin 5, which corresponded to class Elusimicrobia. Bin 9, which corresponded to family Chitinophagaceae belonging to class Bacteroidia, was found only in the sample collected at site 2 in December 2021, whereas bin 10, which corresponded to class Kapabacteria with a family lineage (Palsa-1295), different from family Kapabacteriaceae found in bin 6 was found only in the samples collected at site 2 irrespective of the three months. Bin 1, which corresponded to family Fredricksoniimonadaceae in phylum Omnitrophota, was found in the sample collected at site 3 in November 2021 and those collected at site 2 in the three months. These results indicate that bin cluster at site 1 was very different from those at sites 2 and 3 as also shown in the dendrogram of Fig. 6. It was noted that the total reads mapped were remarkably low at site 3 (Fig. 5).

**Table 4:** Summary of the genomic features and taxonomic characterization of bins for groundwater samples collected from three sites in November and December 2021, and January 2022.

Bin Id	Completeness (%)	Contamination (%)	N50 (bp)	GC (%)	Number of contigs	CheckM marker lineage	Taxonomy-GTDB
bin1	81.0	1.4	4050	56.1	572	k_Bacteria	d_Bacteria; p_Omnitrophota; c_Koll11; o_Zapsychrales; f_Fredricksoniimonadaceae; g_JACQQW01; s_
bin2	81.7	4.1	3281	51.8	1080	o_Cytophagales	d_Bacteria; p_Bacteroidota; c_Bacteroidia; o_Cytophagales; f_Cyclobacteriaceae; g_ELB16-189; s_
bin3	85.4	3.9	8683	65.1	593	c_Alphaproteobacteria	d_Bacteria; p_Pseudomonadota; c_Alphaproteobacteria; o_Micropepsales; f_Micropepsaceae; g_Rhizomicrobium; s_
bin4	96.0	0.4	14,571	64.5	344	c_Alphaproteobacteria	d_Bacteria; p_pseudomonadota; c_Alphaproteobacteria; o_JACAEB01; f_JACAEB01; g_; s_
bin5	78.8	9.8	3019	69.9	958	k_Bacteria	d_Bacteria; p_Elusimicrobiota; c_Elusimicrobia; o_UBA1565; f_UBA9628; g_GWA2-66–18; s_GWA2-66–18 sp016194625
bin6	87.5	1.2	4574	57.1	630	k_Bacteria	d_Bacteria; p_Bacteroidota; c_Kapabacteria; o_Kapabacteriales; f_Kapabacteriaceae; g_UBA10438; s_
bin7	59.1	7.0	1453	39.5	2234	p_Cyanobacteria	d_Bacteria; p_Cyanobacteriota; c_Cyanobacteriia; o_Cyanobacteriales; f_Nostocaceae; g_Sphaerospermopsis; s_Sphaerospermopsis kisseleviana_A
bin8	65.9	2.9	1885	42.4	1850	p_Cyanobacteria	d_Bacteria; p_Cyanobacteriota; c_Cyanobacteriia; o_Cyanobacteriales; f_Microcystaceae; g_Microcystis; s_
bin9	62.0	4.9	1651	40.1	1267	p_Bacteroidota	d_Bacteria; p_Bacteroidota; c_Bacteroidia; o_Chitinophagales; f_Chitinophagaceae; g__Sediminibacterium; s_
bin10	65.9	1.6	2166	47.6	1149	k_Bacteria	d_Bacteria; p_Bacteroidota; c_Kapabacteria; o_Palsa-1295; f_Palsa-1295; g_JAFDZW01; s_
bin11	67.7	2.1	1991	40.4	1245	p_Cyanobacteria	d_Bacteria; p_Cyanobacteriota; c_Cyanobacteriia; o_Cyanobacteriales; f_Nostocaceae; g_Raphidiopsis; s_Raphidiopsis raciborskii

**Figure 7:**
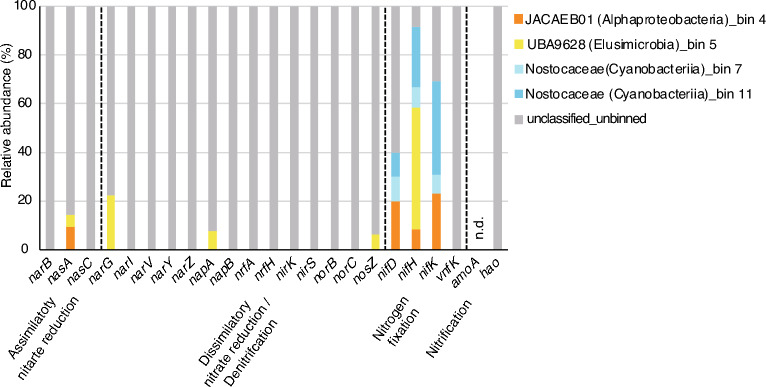
The relative abundance of bacteria harbouring nitrogen metabolic genes for groundwater samples. Taxonomical classification was carried out by the binning of the total gene abundance (TPM) calculated for all groundwater samples collected from sites 1, 2 and 3 each for various nitrogen metabolism genes. The abbreviation "n.d." stands for "not detected".

Figure 7 shows the relative abundance of bacteria harbouring various nitrogen metabolism genes in all groundwater samples from sites 1, 2, and 3. Although most genes involved in nitrate reduction and denitrification could not be binned, a significant portion of the nitrogen fixation genes, including *nifD*, *nifH*, and *nifK* (except for *vnfK*), were assigned to JACAEB01 (Alphaproteobacteria) as bin 4, UBA9628 (Elusimicrobia) as bin 5, and to Nostocaceae (Cyanobacteriia) as bins 7 and 11. A smaller portion of the assimilatory nitrate reduction genes, such as *nasA*, and dissimilatory nitrate reduction/denitrification genes, such as *narG*, *napA* and *nosZ*, were also classified into JACAEB01 (Alphaproteobacteria) and UBA9628 (Elusimicrobia) as bins 4 or 5, respectively.

## Discussion

The topography of the southern part of Okinawa Island is characterized by the Pleistocene Ryukyu limestone terrace, which unconformably overlies the basement mudstone of the Miocene‐to‐Pliocene Shimajiri Group as the surface geology^21^. Groundwater has long been used in this region for agriculture and domestic purposes. In recent years, however, groundwater condition has become serious problem in this area. Actually, the groundwater at site 2 showed a markedly high concentration of NO_3_-N (not less than 20 mg/L, Table 1) and thus was thought to be unsuitable for drinking water purposes because the environmental quality standard in Japan is set to < 10 mg/L. Although the well at site 2 is not used for drinking water, it is located upstream of the drinking water supply. Therefore, the increase in nitrate nitrogen concentration in this local area is of serious concern. In contrast, site 3 tended to have the lowest DO values throughout the year among the three sampling sites^21^. It is noted that site 2 is characterized by a longer distance from the ground surface than that at site 3. DO is considered an indicator of a denitrification response and suggested that site 2 had a mild reducing environment^18^.

Taxonomical analysis based on the 16S rRNA amplicon datasets revealed that class Cyanobacteriia accounted for more than about 20% of the microbial community at site 1, which was markedly different from the other sites (Fig. 2b). The abundancy of Cyanobacteriia was also further confirmed as bins 7, 8 and 11 by assigning taxonomic classification with GTDB-Tk (Fig. 5). Class Kapabacteria was also abundant at site 1 in 16S rRNA amplicon datasets, which was further confirmed by binning (bin 6 in Fig. 6). Class Alphaproteobacteria, abundant at site 2 in 16S rRNA amplicon analysis, was assigned into bins 3 and 4. Sites 2 and 3 showed class Elusimicrobiota to be dominant in 16S rRNA amplicon analysis, which was also assigned as bin 5. Thus, 16S rRNA amplicon and shotgun metagenomic analyses provided the same dominant bacterial classes, demonstrating that the bacterial communities at site 1 were very different from those at sites 2 and 3. These differences among the three sites seem to be reflected by environmental factors as shown in Fig. 3.

The water samples at site 1 were collected from a faucet at the water treatment plant, whereas samples were collected via pumping from observation wells at the other sites. Cyanobacteria may have grown near the faucet from which the water was sampled, and some of the Cyanobacteria may have enter the water during sampling. The second possibility is that the groundwater was contaminated with Cyanobacteria from the surface filtration tank for observation. The growth rate of Cyanobacteria was relatively low^67^, suggesting in this case that this microbe was constantly being mixed with groundwater. It was noted that MetaPhlAn 4 analysis provided many samples with unclassified reads of more than 90%, suggesting that this analysis was not suitable in the present study.

This study demonstrated that various nitrogen metabolism genes were more abundant at site 3 than at sites 1 and 2 (Fig. 4), suggesting that nitrogen metabolism was particularly active at site 3. Although the representative denitrification marker genes, *nirS*/*nirK*^32^*,* were not detected, other denitrification genes and nitrate reduction genes were significantly more abundant at site 3 than those at the other two sites (Fig. 4). This may be related to the fact that the groundwater at site 3 had relatively anaerobic activity, which could be attributed to the low groundwater levels and low DO concentrations, as shown in Table 1. In addition, the high SS and DOC concentrations at site 3 indicate organic matter-driven active nitrogen metabolism in the microbial communities at this site. In addition, site 3 tended to have a high Shannon diversity index (Table 3). The presence of a variety of microorganisms is thought to increase the abundance of nitrogen metabolism genes.

We quantified nitrogen metabolism genes by defining the 'specific abundance of nitrogen metabolism gene' as the product of TPM and the amount of DNA extracted per L of groundwater (ng/L) as shown in Fig. 4. This approach takes into account not only the composition of nitrogen metabolism genes but also the abundance of bacteria in the groundwater. Hence, a high 'specific abundance' of a nitrogen metabolism gene suggests a high abundance of bacteria carrying that gene, indicating active nitrogen metabolism within the groundwater ecosystem. The specific abundances of nitrogen metabolism genes tended to be higher in the samples collected in December 2021 and January 2022 at sites 2 and 3 than those in November 2021(Fig. 4). This was possibly related to the rainfall event that occurred on the day before the samples were collected; the public atmospheric data showed heavy rainfall in December 2021 (Supplementary Table S4). It is plausible to infer that the rain facilitated the leaching of organic matter and nutrients into the groundwater, resulting in increased bacterial proliferation, including bacteria carrying nitrate reduction genes. This environmental condition may have affected the bacterial community.

As shown in Fig. 7, many genes related to nitrate reduction and denitrification could not be assigned, which may be associated with the fact that the groundwater examined in this study contains a high proportion of bacteria with relative abundances below 1% (Fig. 2). However, it is important to note that their presence within the overall microbial community works together for nitrate reduction and denitrification. In contrast, in environments such as soil, bacteria harbouring genes for nitrate reduction and denitrification have been identified^27^, further highlighting the distinctive microbial communities present in groundwater compared to soil. Furthermore, the total reads mapped were remarkably low at site 3 (Fig. 5). These results suggest a unique feature of the microbial community at site 3. Nitrogen metabolism plays very important roles in ecosystems and encompasses very complex pathways^68^. NO_3_-N is one of the most important elements for microorganisms and plants on Earth^69^. NO_3_^−^ is converted to NO_2_^−^ as an intermediate to ultimately produce NH_3_ which consists of assimilatory nitrate reduction and dissimilatory nitrate reduction^70^. It has been reported that *narGHI* and *nasA* genes are the key enzyme genes in the dissimilatory nitrate reduction pathway and assimilatory nitrate reduction pathway, respectively^71^. Interestingly, both genes were found to be abundant at site 3 (Fig. 4). The reaction process changes based on the reaction environment, and various microorganisms contain genes for the respective functions. It is important to understand the comprehensive picture of these processes. However, it was impossible in this study to identify microbial species or genera which may function in nitrogen metabolism. Shotgun metagenomics has been developed^72^, and the data based on the simultaneous analysis of microbial communities and functional genes have been accumulated^73,74^. It is important to isolate or identify groundwater microbes which contain the genes related to nitrogen metabolism for further development of current research.

Further research is needed to verify the correlation between nitrate reduction, denitrification, and other functions that consume NO_3_-N in groundwater to prevent nitrate pollution.

### Supplementary Information

Supplementary Information.

## Data Availability

The DNA sequence obtained during the current study are available in the DDBJ Sequence Read Archives under the accession numbers DRA017557 and DRA015527.
