# Supplementary material for: Metagenomic analysis of the microbial communities and associated network of nitrogen metabolism genes in the Ryukyu limestone aquifer
Source: Sci Rep. 2024 Feb 22;14:4356. doi: 10.1038/s41598-024-54614-8 (PMC10883930; doi:10.1038/s41598-024-54614-8)
Supplement: Supplementary file 1 — Supplementary Information. [file 41598_2024_54614_MOESM1_ESM.pdf]

Metagenomic analysis of the microbial communities and associated network of nitrogen metabolism genes in the Ryukyu limestone aquifer

Rio Maruyama[1], Ko Yasumoto[1], Nanami Mizusawa[1], Mariko Iijima[2], Mina Yasumoto-Hirose[3], Akira Iguchi[2], Oktanius Richard Hermawan[4], Takahiro Hosono[5,6], Ryogo Takada[7], Ke-Han Song[8], Ryuichi Shinjo[8,9], Shugo Watabe[1], Jun Yasumoto[10]*

[1]Kitasato University School of Marine Biosciences, 1-15-1 Kitasato, Minami, Sagamihara, Kanagawa 252-0373, Japan
[2]National Institute of Advanced Industrial Science and Technology (AIST), Tsukuba Central 7, 1-1-1 Higashi, Tsukuba, Ibaraki, 305-8567 Japan
[3]Tropical Technology Plus, 12-75 Suzaki, Uruma, Okinawa 904-2234, Japan
[4]Earth and Environmental Science, Graduate School of Science and Technology, Kumamoto University, 2-39-1, Kurokami, Kumamoto 860-8555, Japan
[5]Faculty of Advanced Science and Technology, Kumamoto University, 2-39-1 Kurokami, Kumamoto 860-8555, Japan
[6]International Research Organization for Advanced Science and Technology, Kumamoto University, 2-39-1 Kurokami, Kumamoto 860-8555, Japan
[7]Center for Strategic Research Projects, University of Ryukyus, Nishihara, Senbaru, Okinawa 903-0213, Japan
[8]Department of Physics and Earth Sciences, University of the Ryukyus, Senbaru–1, Nishihara, Okinawa 903–0213, Japan
[9]Research Institute for Humanity and Nature, 457-4 Motoyama, Kamigamo, Kita-ku, Kyoto, 603-8047, Japan
[10]Faculty of Agriculture, University of the Ryukyu, 1 Senbaru, Nishihara, Nakagami, Okinawa 903-0213, Japan.

*Corresponding author (yasumoto@agr.u-ryukyu.ac.jp)

Supplementary Table S1 Details of the 16S rRNA amplicon datasets at sites 1, 2 and 3 in November and December 2021, and January 2022

Sampling site	Collection date	Obtained sequences count	Post QC sequences count	Post QC bp count	Post QC minimum bp count	Post QC average bp count	Post QC maximum bp count
Site 1	Nov.2021	378,967	241,471	98,851,773	50	409.4	513
	Dec.2021	363,567	234,180	95,829,581	50	409.2	517
	Jan.2022	457,837	225,906	92,326,094	50	408.7	514
Site 2	Nov.2021	507,514	334,671	135,897,550	50	406.1	517
	Dec.2021	339,797	219,042	89,508,574	50	408.6	511
	Jan.2022	373,299	242,214	99,143,986	50	409.3	511
Site 3	Nov.2021	416,879	248,157	101,543,078	50	409.2	515
	Dec.2021	373,584	235,121	96,292,198	50	409.5	516
	Jan.2022	457,837	295,654	120,917,271	50	409.0	516

Supplementary Table S2 Details of the WGS datasets at sites 1, 2 and 3 in November and December 2021, and January 2022

Sampling site	Collection date	Obtained sequences count	Post QC sequences count	Post QC bp count	Post QC minimum bp count	Post QC average bp count	Post QC maximum bp count
Site 1	Nov.2021	1,038,312	879,214	213,987,095	50	243.4	580
	Dec.2021	1,320,792	1,024,895	245,539,249	50	239.6	580
	Jan.2022	1,544,282	1,303,470	275,319,327	50	211.2	580
Site 2	Nov.2021	818,060	656,630	153,712,105	50	234.1	580
	Dec.2021	983,092	714,217	204,504,272	50	286.3	580
	Jan.2022	1,387,233	1,070,412	235,106,701	50	219.6	580
Site 3	Nov.2021	1,257,735	1,046,058	213,506,451	50	204.1	580
	Dec.2021	1,219,300	921,153	235,236,916	50	255.4	580
	Jan.2022	1,220,215	968,157	239,038,342	50	246.9	580

Supplementary Table S3 The correlation coefficients among environmental factors showing low collinearity

	pH	ORP	DOC	HCO_3^-	SS	T-P	NO_3-N	SO_4^{2-}
pH	1.00	0.14	-0.60	-0.70	-0.30	0.19	-0.45	-0.60
ORP	0.14	1.00	-0.47	-0.48	-0.67	0.00	0.29	-0.07
DOC	-0.60	-0.47	1.00	0.45	0.58	0.29	-0.09	0.15
HCO_3^-	-0.70	-0.48	0.45	1.00	0.28	-0.40	0.46	0.80
SS	-0.30	-0.67	0.58	0.28	1.00	0.55	-0.23	0.01
T-P	0.19	0.00	0.29	-0.40	0.55	1.00	-0.60	-0.59
NO_3-N	-0.45	0.29	-0.09	0.46	-0.23	-0.60	1.00	0.88
SO_4^{2-}	-0.60	-0.07	0.15	0.80	0.01	-0.59	0.88	1.00

Dataset is shown in Table 1.

Supplementary Table S4 Precipitation data one week prior to sampling date at sites 1, 2 and 3 in November and December 2021 and January 2022

Sampling date	Before sampling days ago	precipitation (mm/day)
Nov.2021	7	0
	6	4
	5	5
	4	0
	3	0
	2	0
	1	0
	0	0
Dec.2021	7	1.125
	6	0
	5	0
	4	0
	3	0
	2	0
	1	37.5
	0	13
Jan.2022	7	2
	6	0
	5	36
	4	5
	3	0
	2	0
	1	19.5
	0	53

Data from Itokazu City, Okinawa, Japan. https://tenki.jp/amedas/10/50/91241.html

Supplementary Fig. S1a

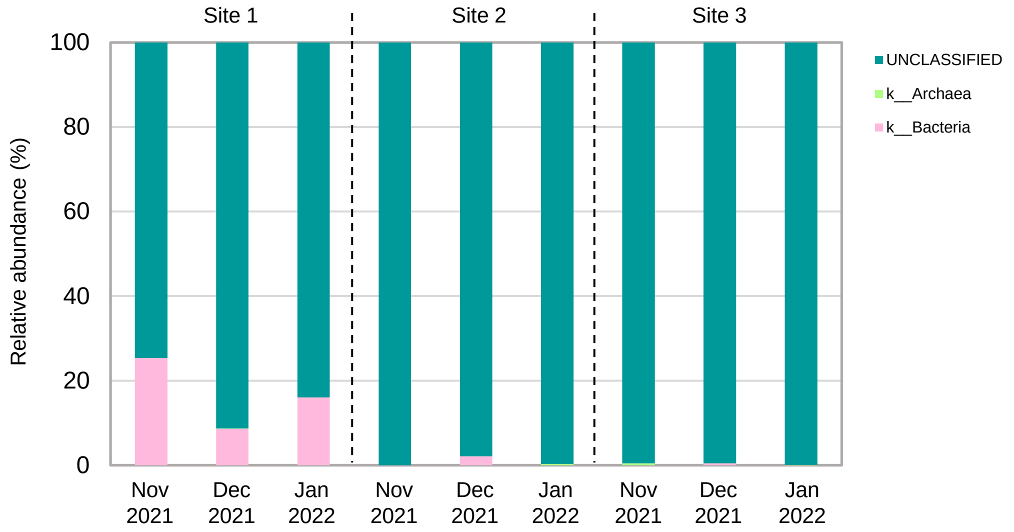

Supplementary Fig. S1b

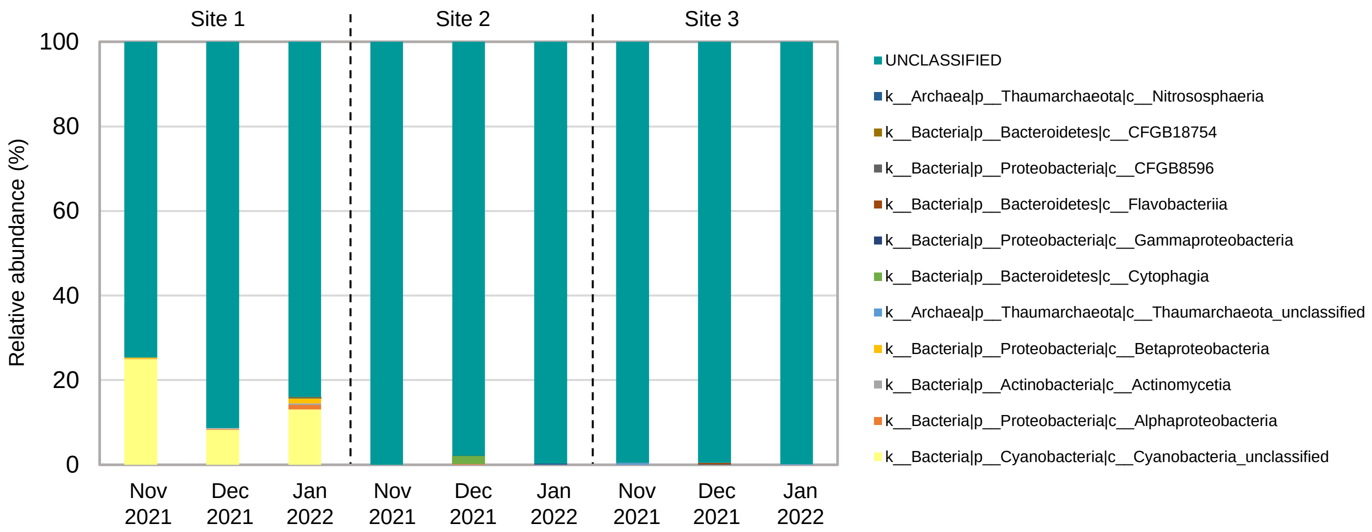

Supplementary Fig. S1c

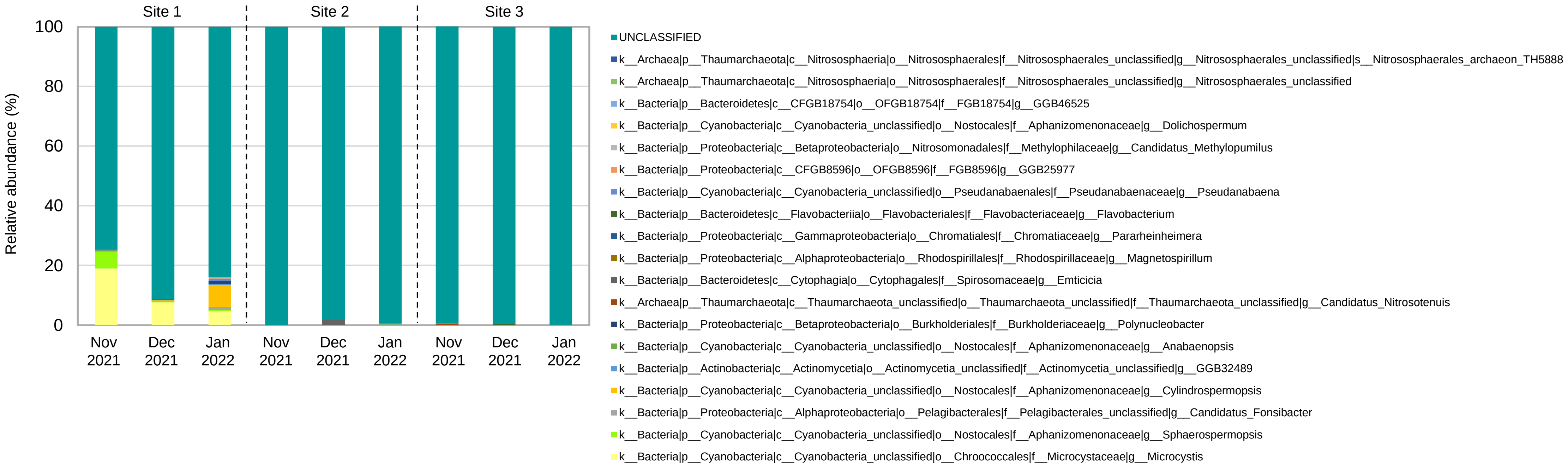

Supplementary Figure S1 The relative abundances of microbes annotated for the WGS reads by MetaPhlAn4 at the domain (a), class (b) and genus (c) levels for groundwater samples collected from the Ryukyu limestone aquifer in November and December 2021 and in January 2022.
